# Data Mining and Fusion Framework for In-Home Monitoring Applications

**DOI:** 10.3390/s23218661

**Published:** 2023-10-24

**Authors:** Idongesit Ekerete, Matias Garcia-Constantino, Christopher Nugent, Paul McCullagh, James McLaughlin

**Affiliations:** 1 School of Computing, Ulster University, Belfast BT15 1ED, UK; 2 School of Engineering, Ulster University, Belfast BT15 1ED, UK

**Keywords:** sensing solution, thermal sensor, Radar sensor, sensor fusion, data mining, in-home, machine learning

## Abstract

Sensor Data Fusion (SDT) algorithms and models have been widely used in diverse applications. One of the main challenges of SDT includes how to deal with heterogeneous and complex datasets with different formats. The present work utilised both homogenous and heterogeneous datasets to propose a novel SDT framework. It compares data mining-based fusion software packages such as RapidMiner Studio, Anaconda, Weka, and Orange, and proposes a data fusion framework suitable for in-home applications. A total of 574 privacy-friendly (binary) images and 1722 datasets gleaned from thermal and Radar sensing solutions, respectively, were fused using the software packages on instances of homogeneous and heterogeneous data aggregation. Experimental results indicated that the proposed fusion framework achieved an average Classification Accuracy of 84.7% and 95.7% on homogeneous and heterogeneous datasets, respectively, with the help of data mining and machine learning models such as Naïve Bayes, Decision Tree, Neural Network, Random Forest, Stochastic Gradient Descent, Support Vector Machine, and CN2 Induction. Further evaluation of the Sensor Data Fusion framework based on cross-validation of features indicated average values of 94.4% for Classification Accuracy, 95.7% for Precision, and 96.4% for Recall. The novelty of the proposed framework includes cost and timesaving advantages for data labelling and preparation, and feature extraction.

## 1. Introduction

Sensor Data Fusion (SDT) is the combination of datasets from homogeneous or heterogeneous sensors in order to produce a complementary, cooperative or competitive outcome [1]. Data from multiple sensors can also be fused for better accuracy and reliability [2]. Processes involved in SDT depend primarily on the type of data and algorithms. The processes typically include data integration, aggregation, filtering, estimation, and time synchronisation [1].

### 1.1. Sensor Data Fusion Architectures

**Figure 1:**
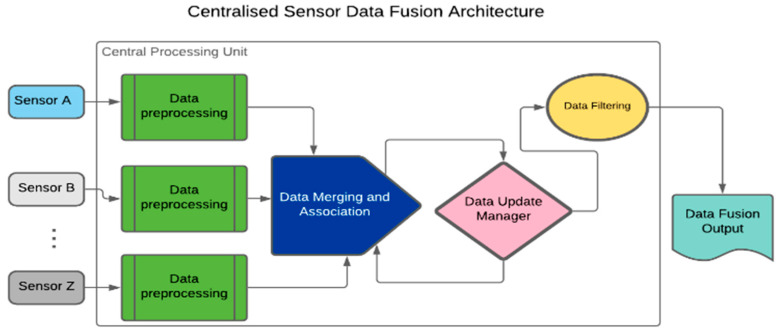
Centralised Sensor Data Fusion architecture outlining the arrangement of processes.

SDT architectures can be categorised into three broad groups, namely centralised, distributed, and hybrid architectures. The centralised architecture is often applied when dealing with homogeneous sensing solutions (SSs) [3]. It involves time-based synchronisation, correction, and transformation of all raw sensing data for central processing. Other steps include data merging and association, updating, and filtering, as presented in Figure 1 [4].

In Figure 1, sensor data are pre-processed in the Central Processing Unit (CPU). The pre-processing procedures entail data cleaning and alignment. The data algorithm requires sub-processes such as data integration, aggregation, and association. Moreover, a Data Update Manager (DUM) algorithm keeps a trail of changes in the output’s status. A DUM is easily implemented in a centralised architecture because of the availability of all raw data in the CPU. Filtering and output prediction follow the data merging and association.

**Figure 2:**
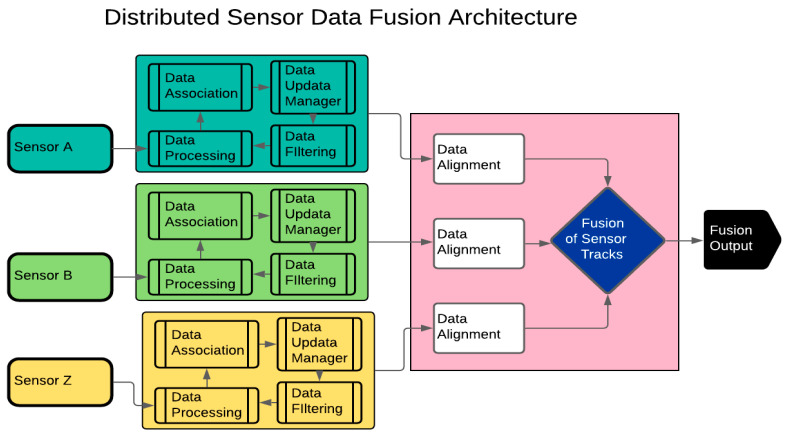
Distributed Sensor Data Fusion architecture showing pre-processing of sensors’ data before filtering and fusion of sensors’ tracks.

In a distributed SDT architecture, data pre-processing for each sensor takes place separately before the actual fusion process, as presented in Figure 2. Unlike with the centralised architecture, gating, association, local track management, filtering, and prediction are performed locally for each sensor before the fusion of the local tracks (Figure 2) [5]. This architecture is best suited for heterogeneous sensors with dissimilar data frames such as datasets from infrared and Radar sensors [6]. Data filtering for each sensor associated with the distributed SDT architecture can be performed by a Kalman Filter (KF) and extended KF [7].

The hybrid SDT architecture unifies the attributes of centralised and distributed architectures. Their capabilities depend on computational workload, communication, and accuracy requirements. The hybrid SDT also has centralised architecture characteristics, such as accurate data association, data tracking, and direct logic implementation. Nevertheless, it is complex and requires high data transfer between the central and local trackers compared with the centralised and distributed architectures. SDT architectures can be implemented using machine learning (ML) and data mining (DM) algorithms.

### 1.2. Data Mining Concepts

DM is an iterative process for exploratory analysis of unstructured, multi-feature, and varied datasets. It involves the use of machine learning, deep learning, and statistical algorithms to determine patterns, clusters, and classes in a dataset [8]. The two standard analyses with the use of DM tools are descriptive and predictive [9]. Whilst descriptive analysis seeks to identify patterns in a dataset, predictive analysis uses some variables in a dataset to envisage some undefined variables [10].

DM can also be categorised into tasks, models, and methods. Tasks-based DM seeks to discover rules, perform predictive and descriptive modelling, and retrieve contents of interest. DM methods include clustering, classification, association, and time-series analysis [11,12]. Clustering is often used in descriptive research, while classification is always associated with predictive analysis [10].

In DM, there is a slight distinction between classification and clustering. Classification is a supervised machine learning approach to group datasets into predefined classes or labels. On the other hand, clustering involves unlabelled data grouping based on similarities of instances such as inherent characteristics of the datasets [10]. Table 1 presents an overview of classification and clustering techniques.

Data clustering techniques such as partition-based, model-based, grid-based, density-based and hierarchical clustering can be used for data grouping [8]. Whilst the density-based approach is centred on the discovery of non-linear structures in datasets, model- and grid-based methods utilise neural networks and grids creation, respectively. The Hierarchical Clustering Technique (HCT) involves the structural representation of datasets as binary trees based on similarities of instances. The HCT also accommodates sub-clusters in nested arrangements. The two main approaches in the HCT are division and agglomeration [24].

The Partitioning Clustering Technique (PCT) groups data by optimising an objective function [8]. The PCT is a non-HCT technique that involves partition iterations to improve the accuracy of formed groups. A popular algorithm in PCT is the K-Means++ Algorithm (KMA) [24,25]. The KMA utilises uncovered characteristics in datasets to improve the similarities of instances. It also reduces data complexities by minimising their variance and noise components [25]. 

Recent studies have suggested the use of a DM method known as Classification by Clustering (CbyC) [26] for classifying unlabelled datasets. The CbyC method converges the algorithms used in data classification and clustering techniques for a systematic analysis of datasets. The basis for CbyC is to discover instance similarities instead of class labels, which are normally used in classification techniques. Also, the CbyC technique is an improvement on the traditional data clustering method, which involves pattern discovery and deviations from natural categories. One of the significant advantages of CbyC is that it saves time and cost for data labelling, especially in big data analysis [26]. Although CbyC does not require class labels for its analysis, its outcome (clustered datasets) can be assigned labels for easy exploration. The present work leveraged the CbyC method to perform the clustering of datasets from thermal and Radar SSs with the help of DM and ML algorithms.

One of the main challenges of SDT is how to deal with heterogeneous and complex datasets with different formats [27]. The present work incorporates datasets from both homogenous and heterogeneous SSs with similar and different formats. The novel contributions of this work are fourfold; namely: (i) presentation of online research findings on DM packages such as RapidMiner Studio, Anaconda, Weka, and Orange data mining software version, (ii) homogeneous data analysis involving binary data from thermal sensors with the software packages, (iii) heterogeneous data analysis involving thermal sensors’ binary data and Radar sensors’ datasets such as speed, Range of Motion (RoM), and the Angle of Approach or Retreat (AAR), and (iv) detailed analysis of the proposed SDT framework.

The remainder of the paper is organised as follows. Section 2 discusses related work on the application of SDT, ML, and DM algorithms; Section 3 presents the materials and methods used in this study; Section 4 presents the conceptual and experimental results, and a detailed analysis of the preferred DM software package; Section 5 discusses findings from the study; and Section 6 presents the conclusion of the study.

## 2. Related Work

SDT algorithms and methods have been utilised in many applications ranging from automobiles to healthcare systems. They can be used to design a redundant, reliable, and complementary system with the intent of enhancing the system’s performance [28]. SDT can be multifaceted, involving many representations such as pixels, features, signals, and symbols [28].

### 2.1. Object Detection

Kim et al. [29] proposed a Radar and infrared sensor fusion system for object detection based on a Radar ranging concept, which required the use of a calibrated infrared camera alongside the Levenberg–Marquardt optimisation method. The purpose of using dual sensors in [29] was to compensate for the deficiencies of each sensor used in the experiment. The implementation of the fusion system was performed on a car with magenta and green cross marks as calibrated points positioned at different distances. The performance of this experiment using the fusion of sensor data was rated 13 times better compared with baseline methods. Work in [30] proposed the fusion of LiDAR and vision sensors for a multi-channel environment detection system. The fusion algorithm enabled image calibration to remove distortion. The study indicated improved performance in terms of communication reliability and stability compared with non-fusion-based approaches.

### 2.2. Automobile Systems

In automated vehicles with driver assist systems, data from front-facing cameras such as vision, LiDAR, Radar, and infrared sensors are combined for collision avoidance and pedestrian, obstacle, distance, and speed detection [31]. The multi-sensor fusion enhanced the redundancy of measured parameters to improve safety since measurement metrics are inferred from multiple sensors before actions are taken. A multimodal advanced driver assist system simultaneously monitors the driver’s interaction to predict risky behaviours that can result in road accidents [31]. Other LiDAR-based sensor fusion research included the use of vision sensors to enhance environmental visualisation [32].

### 2.3. Healthcare Applications

Chen and Wang [33] researched the fusion of an ultrasonic and an infrared sensor using the Support Vector Machine (SVM) learning approach. The study used SDT to improve fall detection accuracy by more than 20% compared with a stand-alone sensor on continuous data acquisition. Kovacs and Nagy [34] investigated the use of an ultrasonic echolocation-based aid for the visually impaired using a mathematical model that allowed the fusion of as many sensors as possible, notwithstanding their positions or formations. Huang et al. [35] proposed the fusion of images from a depth sensor and a hyperspectral camera to improve high-throughput phenotyping. The initial results from the technique indicated more accurate information capable of enhancing the precision of the process. Other studies on the fusion of depth with other SSs can be found in [36,37,38]. The work in [39] involved gait parameters’ measurement of people with Parkinson’s disease, by the fusion of depth and vision sensor systems. An accuracy of more than 90% was obtained in the study. Also, in Kepski and Kwolek [40], data from a body-worn accelerometer was fused with depth maps’ metrics from depth sensors to predict falls in ageing adults. The proposed method was highly efficient and reliable, showing the added advantages of sensor fusion. Work in [41] proposed the fusion of an RGB-depth and millimetre wave Radar sensor to assist the visually impaired. Experimental results from the study indicated the extension of the effective range of the sensors and, more importantly, multiple object detection at different angles.

### 2.4. Cluster-Based Analysis

The integration of SDT algorithms with ML and DM models can help predict risky behaviours and accidents [33,42,43,44,45]. Work in [46] discussed the use of Cluster-Based Analysis (CBA) for a data-driven correlation of ageing adults that required hip replacement in Ireland. Experimental results from the study suggested three distinct clusters with respect to patients’ characteristics and care-related issues. In [47], data evaluation using CBA helped in clustering healthcare records such as illness and treatment methods. A combined method, including CBA for user activity recognition in smart homes, was proposed in [48]. Experimental results indicated higher probabilities for activity recognition owing to the use of a combination of a K-pattern and artificial neural network. Work in [49,50,51] proposed the use of the CBA method in health-related data analysis. Experimental results indicated the suitability of the method for pattern identification and recognition in datasets.

The present work considers a cluster-based data fusion technique with the help of DM software packages, namely, RapidMiner Studio (RMS), Weka DM Software (WDMS) and Orange DM Software (ODMS). The rationale for using these packages includes their analytical data workflows, interactive data visualisation, and predictive capabilities. Other attributes include the ability of their algorithms to discover patterns in binary images, unsupervised learning capabilities, ease of use, and the integration of ML algorithms [52,53].

## 3. Materials and Methods

The methods used in our work include, first, conceptual and evaluation methods to select a suitable software package from a list including WDMS, RMS, Anaconda, and ODMS; second, data analysis using the software packages; third, a detailed description and evaluation of the proposed framework.

Whilst the conceptual methodology informed the initial selection of software packages for the research, the experimental methodology was adopted for testing with real data obtained during sprained ankle rehabilitation exercises. The basis for the preliminary consideration of the software packages included the ability to recognise and categorise binary images, unsupervised feature extraction [54], CbyC capabilities [22,24,26], and ease of data fusion. The experimental methodology involved data collection processes with the aid of single, homogeneous, and heterogeneous SSs. 

Qualitative data [55,56,57] such as postural orientations and actions in the form of binary images acquired with a thermal SS were utilised in this work. The rationale for using binary images for this study was to protect the privacy of occupants. Further, binary images posed peculiar challenges in the implementation of AI in healthcare datasets [58,59] when compared with RGB and greyscale images. Therefore, the ability of a software package to perform CBA with binary images was one of the requirements for suitability to this framework. Likewise, binary images were considered suitable given they require less storage space [60].

Data gleaned from the Radar and the Infrared Thermopile Array (ITA) thermal sensors were analysed using the selected software packages, namely, RMS, WDMS, and ODMS. The WDMS is a Java-based package, whilst RMS and ODMS are Python-oriented. DM and ML models such as Random Forest (RF), Decision Tree (DT), AdaBoost, Logistic Regression (LR), Support Vector Machine (SVM), Stochastic Gradient Descent (SGD), and Naïve Bayes were used to compute the Classification Accuracy (CA) metrics from the packages. First, the rationale for choosing these algorithms was because of their availability in all three software packages. Second, those not available in all three were considered based on their capability for image-based CBA. Whilst binary images were obtained from a 32 by 32 thermal sensor, speed, RoM, and AAR metrics, recorded during Lower Extremity Rehabilitation Exercises (LERE) monitoring, were obtained from a Radar sensor. Both the thermal and the Radar sensors generated timestamps that were used as a basis for the data fusion. The study aimed to (i) perform a CbyC on homogeneous and heterogeneous datasets using selected software packages, (ii) rate the accuracy metrics of the packages using ML algorithms, and (iii) evaluate the software packages based on their ease of use and feature extraction capabilities, amongst other factors. The experimental procedure was considered in two iterations, namely homogenous and heterogeneous iterations. The rationale for the homogenous iteration was to examine the performance of the packages on datasets from similar sensors positioned at different locations within a lab setting which mimics the home environment. The latter iteration, heterogeneous, tested their performance on dissimilar SSs also placed at different locations within the environment.

The homogenous iteration included the thermal images gleaned from lateral and ceiling ITA thermal SSs. In this pathway, 574 binary images from each ITA sensor were used. These images were sorted based on their timestamps. Three software packages (ODMS, WDMS, and RMS) were used to analyse the images. Moreover, cluster-based 10-fold cross-validation and prediction were performed on the images using DM and ML models.

**Figure 3:**
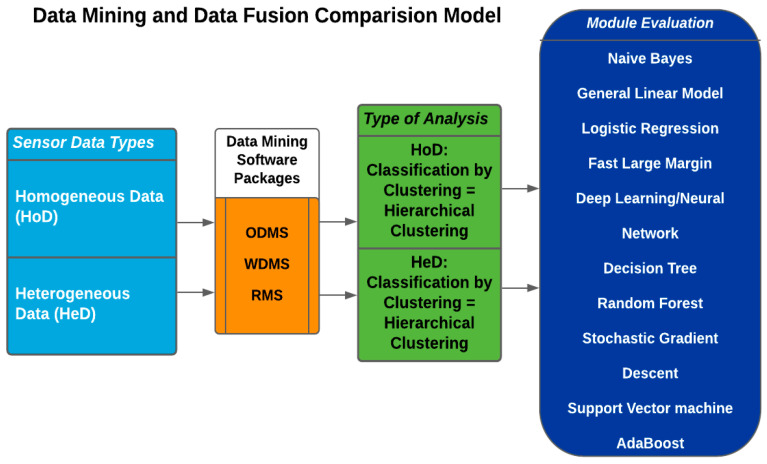
Data mining and fusion comparison model. ODMS = Orange Data Mining Software, RMS = RapidMiner Studio, WDMS = Weka Data Mining Software, HoD = homogenous datasets, HeD = heterogeneous datasets.

The heterogeneous iteration entailed 574 binary images and 1722 (574 rows × 3 columns) Radar sensor datasets. Image pre-processing, which entailed image resizing and normalisation, was performed on MATLAB and aggregated on a bespoke time-series module referred to as Sensor Central [61]. The datasets were uploaded to the software packages through their respective data import interfaces. Whilst heterogeneous dataset fusion using WDMS was challenging with their Java-based algorithm, the process was seamless using the ODMS package. A workflow of the comparative consideration is presented in Figure 3.

In Figure 3, the binary images were uploaded to the ODMS workbench directly from folders and sub-folders. Contrarywise, WDMS requires them to be uploaded in a CSV, ARFF, etc., file format. Preparing these files by hand takes a lot of time; however, with the help of the MATLAB application or ODMS, the information was easily extracted from image folders. In the same vein, RMS required a CSV or other type of file rather than a direct image upload from folders. The data upload process underscores one of the advantages of ODMS over WDMS and RMS.

Feature extraction from the binary images for all the packages was performed in ODMS. This is because the generic features generated by WDMS and RMS did not contain details such as image size, width, height, and other distinguishing metrics necessary for a proper CBA. Also, whilst it was possible to extract ten generic features only from the WDMS at each instance, 1000 features were extracted from each binary image in addition to image length, width, and height from the ODMS package.

## 4. Results

Results from this study are presented in fourfold as follows: (i) conceptual findings, (ii) homogeneous experimental analysis, (iii) heterogeneous experimental analysis, and (iv) detailed description and analysis of the proposed SDT framework.

### 4.1. Conceptual Findings

Research findings by Predictive Analysis Today (PAT) [62] presented DM tools ratings based on their ease of use, performance index, functionality and feature management, availability of advanced features, and user experience. The top-rated four are presented in Table 2.

From Table 2, ODMS has the highest average rating of 94.2%, followed by RMS, 90.2%, and WDMS, 88.2%. Anaconda is rated the least with 77.2%; hence, it was not considered for further data analysis in this study. Whilst RMS has the best rating in terms of its functionality at 96%, ODMS and WDMS were rated 95% and 92% for functionality, respectively. Ease of use and user implementation were the best rated in ODMS at 96% and 90% compared with other packages.

### 4.2. Homogeneous Data Analysis

The initial observation indicated that data fusion tools such as merge and union performed well in ODMS and RMS, respectively. WDMS and RMS, however, were unable to work with the data directly. Hence, their data were arranged in a CSV file before being analysed on their respective platforms. Moreover, a 10-fold cross-validation CbyC was performed on the data following normalisation using the DM models. 

The CA from the first iteration involving homogeneous data fusion is presented in Table 3.

The results presented in Table 3 show that ODMS has an average accuracy of 84.7%, followed by WDMS, 76.2%, and RMS, 72.0%. A further breakdown of the results shows that ODMS has the highest accuracy of more than 94.0% in four models. RMS and WDMS, however, scored less than 90.0% in all their models. The performance of these models in different software packages was attributed to the number of inherent computational resources that were available in the packages. This property of a model is referred to as model efficiency. Hence, the LR, SGD, SVM, and NN models were very efficient at employing ODMS for processing binary data such as was used in this study.

### 4.3. Heterogeneous Data Analysis

Metrics such as Naïve Bayes, Generalised Linear Model, Fast Large Margin, amongst others, were used for the heterogeneous data analysis. A detailed breakdown of the data import process is presented in Section 4.4. The accuracy values of the models are presented in Table 4.

From Table 4, ODMS has the highest average accuracy of 95.7%, while WDMS and RMS had 64.7% and 59.7% accuracies, respectively. DT and CN2 Induction obtained 99.5% accuracy each in ODMS. CN2 Induction is an algorithm that is designed to classify an imperfect set of data [45]. Also, while the lowest accuracy value was 80.7% in ODMS, the highest accuracy values in WDMS and RMS were 70.0% and 62.2%, respectively. Due to the many advantages of ODMS (as presented in this study and other relevant literature [45]), the SDT framework proposed in this work leveraged the ODMS package.

### 4.4. Proposed Data Fusion Framework

**Figure 4:**
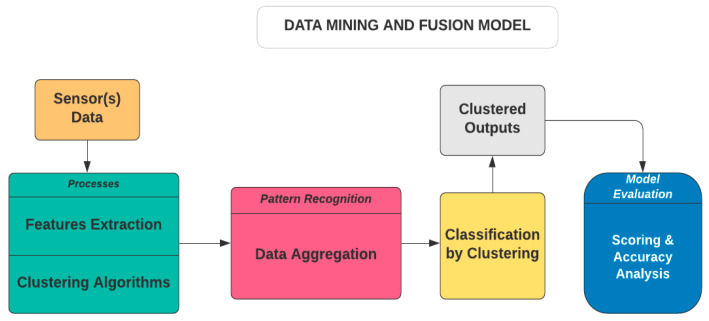
Data mining and fusion model indicating the processes involved from data acquisition to model evaluation.

ODMS is an open-source data analytics and visualisation tool. It is based on the visual design layout and Python scripting. It consists of DM and ML algorithms that extend its functionality. The data component layout consists of a file toolkit, CSV file import, pivot table, Python script, and datasets toolkits. It can be used for distributed CbyC processes, which are fundamentally based on HCA and KMA. A simplified description of the proposed framework is presented in Figure 4.

**Figure 5:**
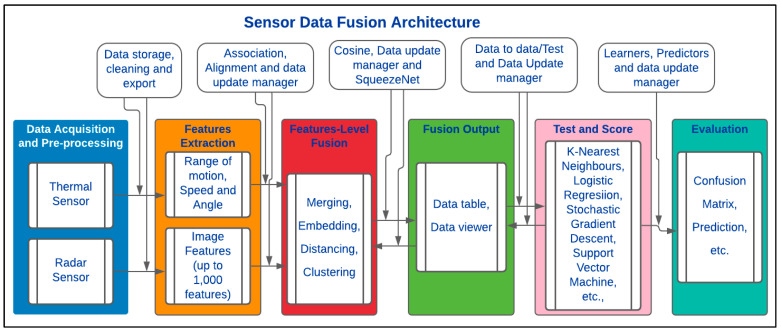
Detailed Sensor Data Fusion architecture based on Orange Data Mining Software package for homogeneous and heterogeneous datasets.

In Figure 4, the data processing unit takes inputs from the sensor(s) before feature extraction and aggregation. This is then followed by training the CbyC algorithms on the datasets. The clustered outputs are evaluated to ascertain the accuracy of the clustered entities using several classification algorithms. In addition, the model can analyse and fuse both homogeneous and heterogeneous datasets without rigorous data labelling processes. A more detailed description of this model is presented in Figure 5.

In Figure 5, data acquisition and pre-processing are performed by individual sensors: Radar and thermal. For Radar sensors, signal strength, RoM, speed, and AAR are acquired and are stored in a CSV file. Parameters such as time, range, speed, and AAR are extracted from the Radar sensor, while up to 1000 features are extracted from the thermal (greyscale and binary) images. Thermal blobs gleaned from the ITA sensor are stored in a predetermined folder with timestamps. The rationale for storing the data from both sensors with timestamps is to enable a time-based fusion of the data.

Furthermore, data from the sensors are exported to a DM and fusion block using file import and image import toolkits. While the former enables the reading of tabular data and their instances from a spreadsheet or a text document, the latter helps upload images from folders. Information such as image width, size, height, path, and name are automatically appended to each image uploaded in a tabular format.

Preliminary feature extraction can be programmed to begin automatically or with a click at the data merging component. A matching-row-append, matching-rows-pairs function or concatenation is used to ensure that the features are correctly matched. Definitive feature extraction takes place at a data embedding capsule where more than 1000 features, represented as vectors (n_0_ to n_999_), are extracted from each ITA image. Feature extraction can be performed by using deep learning image embedders for image recognition such as painters, Inception v3 (IV3), deepLoc, squeezeNet, and Convolutional Neural Networks (CNN) [63]. The rationale for using these embedders includes their efficient and distributed training processes [64].

Metrics, namely Euclidean, cosine, Manhattan, Jaccard, Spearman, and Pearson, are situated in the Distances Application (DA). A feature normaliser, which performs column-wise normalisation for both categorical and numerical data, can be applied to both homogeneous and heterogeneous datasets [63]. The output of the DA is connected to the HCA for the classification of the distanced features. Moreover, a dendrogram corresponding to a cluster of similar features from the DA is computed using the HCA. Other DA-based features used include weighted, average, single, or complete associations of data. 

**Figure 6:**
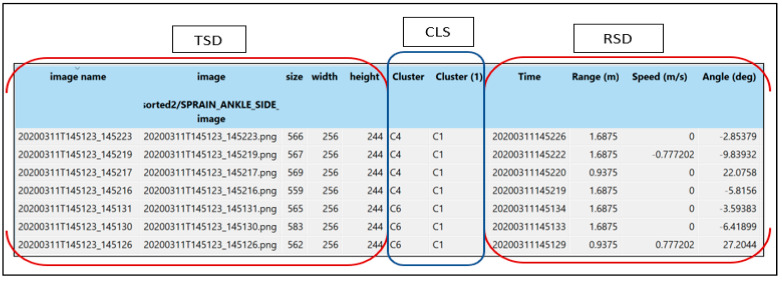
Data table showing combined data from ITA and Radar sensors. TSD = thermal sensor data, CLS = clusters, and RSD = Radar sensor data.

The Louvain clustering algorithm can be used to detect and integrate communities into the module. It can also be utilised for grouped feature conversion into a K-Nearest Neighbours (KNN) graph and structures optimisation to obtain nodes that are interconnected. The principal graph parameters of Louvain clustering are KNN, resolution, and distance metrics [65]. Figure 6 presents a data table after data fusion where image name, path, size, width, clusters, timestamps, RoM, speed, AAR, and up to 1000 features can be viewed.

**Figure 7:**
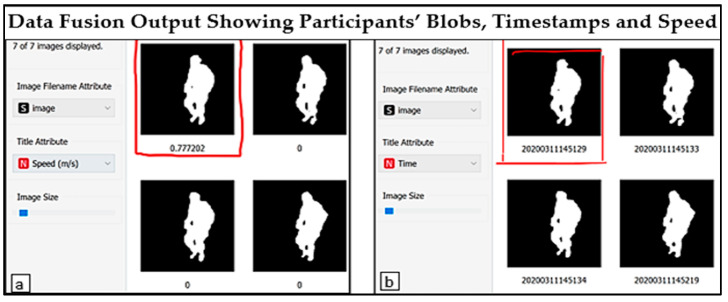
Data viewer interface showing data fusion output. (**a**) Side view of interface showing speed during Lower Extremity Rehabilitation Exercise (LERE), and (**b**) side view of interface showing timestamp during LERE. The highlighted parts in (**a**,**b**) indicate the speed and timestamps in which the exercises were performed, respectively, after data fusion.

In Figure 6, the areas marked as TSD, CLS and RSD represent thermal, clusters and Radar sensor data, respectively. Moreover, the first two columns of TSD indicated the timestamps, which also represent the image name. These are followed by size, width, and height. The clusters of the images are labelled as CLS (Figure 6). Data from the Radar sensor are represented by the time, RoM, speed, and angle in the area marked RSD (refer to Figure 6). Similarly, the data viewer toolkit can be used to visualise images (after fusion) and relevant information such as speed, RoM, and the AAR of participants from the selected cluster(s), as presented in Figure 7a,b.

The side view of participants performing LERE in a laboratory sitting room that mimics a real-life sitting room is presented in Figure 7a,b. The results indicated the action that was taken at a particular time interval. Hence, activities with similar features are grouped in clusters, thus enabling the visualisation of similar activities notwithstanding the day or time when they were performed. In Figure 7a, the speed at which the exercise was performed is appended to the image as 0.777202 m/s, as indicated on the top left image. On the other hand, the time at which the exercise was performed is appended to the top left image (Figure 7b) as 20200311145129 (11 March 2020 at 29s past 14.51). With these data fusion outputs, tangible information that can help exercise prescription by therapists can be obtained.

Evaluation of the clustering accuracy of the detailed SDT (Figure 5) can be performed using cross-validation, Test on Train Data (TTD), or random sampling techniques. Cross-validation is a sampling technique used for the evaluation of models by training them on a fraction of the input data [66]. Comparative results from the same datasets based on cross-validation and TTD techniques are presented in Table 5 and Table 6, respectively.

In Table 5, cross-validation by features was performed on the 574 ITA-32 images and 1722 Radar sensor data using DM algorithms such as RF, NN, KNN, and CN2 Induction. These algorithms were chosen at random for the comparison of the cross-validation and TTD sampling techniques. From the evaluation, RF has the least value for Area Under the Curve (AUC), followed by CN2 Induction. CA was, however, higher with NN, followed by RF and then KNN and CN2. Also, the value of the weighted average (F1) [63] was higher (more than 94%) with NN in Precision, Recall, and Specificity.

TTD implies using all the data for both training and testing. In most instances, TTD can give incorrect results, and as such, it is not a recommended evaluation technique. The evaluation accuracies for the models using the TTD technique are presented in Table 6.

As presented in Table 5, the results of all the models are higher in TTD than in the cross-validation technique (Table 6). For example, RF, which was 85.2% in the cross-validation technique, attained an accuracy of 100.0% in TTD. Similarly, CN2 Induction, which was 85.2% with cross-validation (Table 5), attained an accuracy of 100% (Table 6).

## 5. Discussion

The present work on SDT using DM and ML models leveraged ODMS for feature-level fusion using a matching-row-append, matching-rows-pairs function or concatenation of features. The framework suits both homogeneous and heterogeneous datasets ranging from RGB to greyscale and binary images.

### 5.1. The Proposed Framework vs. Others

Experimental results indicated that our proposed framework has a better performance than the SDT frameworks in [6,67] in terms of the accuracy metrics. In [68], a multimodal sensor fusion framework was used to estimate the states of dynamic legged robots using heterogeneous datasets from wearables such as a gyroscope and accelerometer. The added advantage of our work includes the performance of homogeneous data analysis and the use of unobtrusive (non-wearable) SSs. 

Furthermore, the proposed framework contains evaluation modules for testing and scoring the output of the data fusion and classification of features, as presented in Figure 5. It also offers visualisation toolkits at every stage to help evaluate the outcomes of the fusion processes.

### 5.2. Advantages of the Proposed Framework

Our proposed framework offers advantages such as the ability to discover patterns in binary images, unsupervised learning capabilities, ease of use, and the integration of ML algorithms. It also presents a range of flexibilities depending on the type of sensors used and expected results. As an example, a scatter plot, data distribution toolkit, or heat map can be included in the framework depending on the intent of the user. Other algorithms which can also be featured in the architecture include data randomisation, ranking, transposition, and correlation.

This framework addresses the drawbacks of the centralised architecture, such as computational overload. It entails the modification of the generic distributed SDT (earlier described). Its main advantages include (i) communication adaptability, (ii) lesser computational load due to distributed functions, (iii) minimal communication delay, and (iv) higher stability due to its shared processes [3]. The framework can be applied to in-home upper and LERE monitoring, activity recognition, fall detection, and ADLs monitoring.

### 5.3. Limitation of the Proposed Framework

The main limitation of the proposed framework is that models such as DT and RF in ODMS perform poorly on homogeneous datasets when computing their AUC. Hence, DT and RF scored 62.3% and 73.9%, respectively, on AUC due to their inability to compute the definite integral datasets. This challenge was mostly experienced with binary datasets.

Furthermore, the low accuracy was also attributed to the inherent characteristics of the algorithms including their signal-to-noise ratio and model efficiency in ODMS [69]. Additionally, the homogenous datasets used in this study were largely binary images that were susceptible to noise. Although efforts were made to reduce the noise using background subtraction algorithms during pre-processing, the presence of noise was unavoidable. Contrariwise, the heterogeneous datasets, which also contained textual datasets obtained from the Radar sensor, performed better with DT and RF, further demonstrating the potential merits of heterogeneous datasets in real-world applications.

## 6. Conclusions and Future Work

This paper proposed an SDT framework for in-home applications. PAT research findings and comparative study on DM software packages such as RMS, WDMS, Anaconda, and ODMS featured ODMS as the preferred DM tool with an average rating of 94.2% based on their ease of use, performance index, functionality and feature management, availability of advanced features, and user experience. An SDT analysis with the proposed framework indicated average accuracies of 84.7% and 95.7% for homogeneous and heterogeneous SDT, respectively. Information obtained from the SDT output can help estimate the speed at which in-home exercises such as post-stroke and LERE were performed. Other details such as the timestamps, the RoM and the AAR can help the therapist determine if recommended activities were performed as prescribed.

Future work will extend the proposed SDT framework to other deep learning models. The application to ambient assisted living activity modelling using other sensing solutions such as pressure floor mats and UWB-based positioning SSs will be considered. It will study the use of pressure floor mats as a gold standard to ascertain the actual locations of room occupants within the home environment. This will aid the detection of abnormal activities during ADLs performance and further support independent living.

## Figures and Tables

**Table 1:** Classification and clustering techniques in data mining.

Classification Techniques	Application of Classification Techniques	Clustering Techniques	Application of Clustering Techniques
Neural Network	E.g., stock market prediction [13]	Partition-based	E.g., medical datasets analysis [14]
Decision Tree	E.g., Banking and finance [15]	Model-based	E.g., multivariate Gaussian mixture model [16]
Support Vector Machine	E.g., big data analysis [17]	Grid-based	E.g., large-scale computation [18]
Association-based	E.g., high dimensional problems [19]	Density-based	Applications with noise. E.g., DBSCAN [20]
Bayesian	E.g., retrosynthesis [21]	Hierarchy-based	E.g., Mood and abnormal activity prediction [22,23]

**Table 2:** Predictive Analysis Today (PAT) research rating of data mining software packages.

Parameters	ODMS (%)	RMS (%)	WDMS (%)	Anaconda (%)
Ease of Use Interface	96.0	94.0	91.0	78.0
Functionality and Features Management	95.0	96.0	92.0	78.0
Software Integration	94.0	95.0	90.0	76.0
Performance Index	95.0	95.0	91.0	77.0
Advanced Features Incorporation	95.0	94.0	92.0	77.0
User Rating on Implementation	90.0	67.0	73.0	77.0
Average Rating	94.2	90.2	88.2	77.2

Legend: ODMS = Orange Data Mining Software, WDMS = Weka Data Mining Software, and RMS = RapidMiner Studio (RMS).

**Table 3:** Comparison of software packages based on classification by clustering method. The accuracies of the machine learning models used for the homogeneous datasets are presented.

Model	ODMS CA (%)	WDMS CA (%)	RMS CA (%)
Naive Bayes	79.9	77.0	80.8
Generalised Linear Model	NA	NA	82.7
Logistic Regression	94.1	74	22.9
Fast Large Margin	NA	NA	83.3
Deep Learning/Neural Network	94.2	NA	86.1
Decision Tree	62.3	77.0	NA
Random Forest	73.9	83.0	55.1
Stochastic Gradient Descent	94.5	71.0	87.1
Support Vector Machine	94.0	75.0	78.3
Average based on Available Models	84.7	76.2	72.0

Legend: ODMS = Orange Data Mining Software, WDMS = Weka Data Mining Software and RMS = RapidMiner Studio (RMS), NA = Not available.

**Table 4:** Comparison of software packages based on classification by clustering method. The accuracies of the machine learning models used for the heterogeneous datasets are presented.

Model	RMS CA (%)	WDMS CA (%)	ODMS CA (%)
Naive Bayes	60.4	67.0	80.7
Generalised Linear Model	60.7	NA	NA
Fast Large Margin	62.2	NA	NA
Deep Learning/Neural Network	59.2	NA	98.9
Decision Tree	54.3	64.0	99.5
Decision table	NA	69.0	NA
Random Forest	59.2	70.0	89.9
Stochastic Gradient Descent	60.1	NA	99.3
Support Vector Machine	61.3	48.0	98.4
K-Nearest Neighbours	NA	NA	99.1
CN2 Induction	NA	NA	99.5
J48	NA	70.0	NA
Average	59.7	64.7	95.7

Legend: ODMS = Orange Data Mining Software, WDMS = Weka Data Mining Software and RMS = RapidMiner Studio (RMS), NA = Not available, CA = Classification Accuracy.

**Table 5:** Evaluation based on the cross-validation of results from data mining models.

Model	AUC (%)	CA (%)	F1 (%)	Precision (%)	Recall (%)	Log Loss (%)
Random Forest	85.2	96.8	96.0	95.8	96.8	0.2
Neural Network	95.5	98.6	98.6	98.6	98.6	0.1
K-Nearest Neighbours	95.5	95.5	94.6	93.7	95.5	0.1
CN2 Induction	87.8	94.6	94.6	94.6	94.6	0.1
Average	91.0	96.4	96.0	95.7	96.4	0.1

Legend: Area Under the Curve (AUC), Classification Accuracy (CA), F1 = weighted average.

**Table 6:** Evaluation based on ‘Test on Train Data’.

Model	AUC (%)	CA (%)	F1 (%)	Precision (%)	Recall (%)	Log Loss (%)
Random Forest	100.0	99.5	99.5	99.5	99.5	0.0
Neural Network	100.0	100.0	100.0	100.0	100.0	0.0
K-Near Neighbours	98.7	98.2	98.0	98.0	98.2	0.1
CN2 Induction	100.0	100.0	100.0	100.0	100.0	0.0

Legend: Area Under the Curve (AUC), Classification Accuracy (CA), F1 = weighted average.

## Data Availability

Not applicable.
